# Role of Tax protein in human T-cell leukemia virus type-I leukemogenicity

**DOI:** 10.1186/1742-4690-1-20

**Published:** 2004-08-13

**Authors:** Inbal Azran, Yana Schavinsky-Khrapunsky, Mordechai Aboud

**Affiliations:** 1 Department of Microbiology and Immunology and Cancer Research Center, Faculty of Health Sciences, Ben Gurion University of the Negev, Beer Sheva 84105, Israel

## Abstract

HTLV-1 is the etiological agent of adult T-cell leukemia (ATL), the neurological syndrome TSP/HAM and certain other clinical disorders. The viral Tax protein is considered to play a central role in the process leading to ATL. Tax modulates the expression of many viral and cellular genes through the CREB/ATF-, SRF- and NF-κB-associated pathways. In addition, Tax employs the CBP/p300 and p/CAF co-activators for implementing the full transcriptional activation competence of each of these pathways. Tax also affects the function of various other regulatory proteins by direct protein-protein interaction. Through these activities Tax sets the infected T-cells into continuous uncontrolled replication and destabilizes their genome by interfering with the function of telomerase and topoisomerase-I and by inhibiting DNA repair. Furthermore, Tax prevents cell cycle arrest and apoptosis that would otherwise be induced by the unrepaired DNA damage and enables, thereby, accumulation of mutations that can contribute to the leukemogenic process. Together, these capacities render Tax highly oncogenic as reflected by its ability to transform rodent fibroblasts and primary human T-cells and to induce tumors in transgenic mice. In this article we discuss these effects of Tax and their apparent contribution to the HTLV-1 associated leukemogenic process. Notably, however, shortly after infection the virus enters into a latent state, in which viral gene expression is low in most of the HTLV-1 carriers' infected T-cells and so is the level of Tax protein, although rare infected cells may still display high viral RNA. This low Tax level is evidently insufficient for exerting its multiple oncogenic effects. Therefore, we propose that the latent virus must be activated, at least temporarily, in order to elevate Tax to its effective level and that during this transient activation state the infected cells may acquire some oncogenic mutations which can enable them to further progress towards ATL even if the activated virus is re-suppressed after a while. We conclude this review by outlining an hypothetical flow of events from the initial virus infection up to the ultimate ATL development and comment on the risk factors leading to ATL development in some people and to TSP/HAM in others.

## Introduction

Human T-cell leukemia virus type-I (HTLV-1) is the first discovered human retroviral pathogen [1]. It has been firmly implicated with the etiology of an aggressive malignancy known as adult T-cell leukemia (ATL) and of a neurological progressive inflammatory syndrome called tropical spastic paraparesis or HTLV-1 associated myelopathy (TSP/HAM). In addition, there are indications that it might be also associated with certain other clinical disorders [2,3]. In culture HTLV-1 can infect a wide variety of cell types from different species. However, in natural human infections this virus targets mainly mature CD4^+ ^helper T-cells [4-6], resulting in benign expansion the infected cells [7]. Clonal or oligoclonal expansion of the infected CD4^+ ^cells is mostly associated with development of ATL and 90–96% of the HTLV-I DNA is, indeed, found to segregate with CD4 cells in the peripheral blood of ATL patients [4], whereas CD4/CD8 double-positive leukemic cells are detected in rare cases [8]. CD8^+ ^T-cells might also be infected [9,10], but their expansion is rather polyclonal and frequently occurs in asymptomatic carriers. Therefore, their disease association is unclear yet [11].

Shortly after infection the virus enters into a latent state, rendering the infected individuals asymptomatic seropositive carriers. About 5% of these individuals develop one of the viral associated diseases 10 to 40 years after infection. During latency the viral gene expression in the peripheral blood lymphocytes (PBLs) of such carriers is very low. Viral RNA is undetectable by Northern blot analysis in most of the infected cells (i.e. viral DNA harboring cells) freshly isolated from their peripheral blood [5], although it can be detected in some carriers by the highly sensitive RT/PCR analysis [12]. Furthermore, very little or no viral proteins are detectable in the carriers' PBLs [12,13]. Notably, despite this low virus expression, healthy carriers contain antibodies against viral antigens. They also display anti HTLV-1 specific cytotoxic T-lymphocytes (CTL) activity at variable levels that seem to be determined by hosts' genetic determinants, particularly by those associated with their HLA antigens [3,14,15]. Experimental evidence has been reported, pointing to the critical role of these two anti HTLV-1 immune response arms in keeping this low viral expression. It has been repeatedly shown that PBLs isolated from such carries start eliciting high viral gene expression within few hours of growing in culture [10,13,16]. However, Tochikura et al. have noted that addition of sera from HTLV-1 carriers or patients to the culture medium reduces this viral expression at an efficiency which correlates to their titer of anti HTLV-1 antibodies and that removal of these antibodies by protein A abolishes this inhibition. No such inhibition has been observed with sera of uninfected control donors [13]. Other workers have analyzed the level of HTLV-1 expression in PBLs grown in whole blood samples of various infected individuals and found that depletion of CTLs from these samples remarkably increases in the number of virus-expressing CD4+ cells compared to that found in the same samples without CTL-depletion [10,16]. Furthermore, these authors have demonstrated a similar increase by blocking the CTL-mediated cytolytic activity with concanamycin A. These data strongly suggest that anti HTLV-1 CTL activity, mounting in infected individuals, eliminate cells with high level of viral antigens and keep, thereby, the overall virus expression in the carriers' PBLs at low level. In view of this low virus expression, the viral load in HTLV-1 infected individuals has been noted to expand primarily through proliferation of the proviral DNA-harboring cells rather than through repeated cycles of cell-to-cell infection of new uninfected cells [17]. As discussed in our recent review article [18], this expansion pattern is widely considered to account for the maintenance of high sequence stability of the viral genome throughout the hundreds of thousands years of evolution since its emergence from its simian T-lymphotropic retrovirus origin. This stability is in striking contrast to the high genetic diversity of HIV-1 which is known to spread within the infected individuals through repeated infections of new cells by cell-free virions [19].

Although the mechanism of HTLV-1 pathogenicity is not fully understood yet, it is widely believed that a virally encoded transactivator protein, called Tax, plays a central role in this mechanism. It should, therefore, be noted that while the low level of the virus gene expression detected in latently infected carriers might be sufficient for maintaining their anti HTLV-1 seropositivity and CTL activity, the low Tax level, resulting from this reduced viral expression is, most likely, below its pathogenic threshold. This implies that generating an HTLV-1 related disease requires an activation of the dormant virus in order to elevate Tax to its pathogenic level.

In this article we present a comprehensive review of the wide range of Tax molecular interactions and biological effects that might be closely relevant to the mechanism of ATL genesis and summarize this information by proposing hypothetical flow of a stepwise pathway leading to this malignancy or to TSP/HAM.

### HTLV-1 genomic structure and gene expression

**Figure 1:**
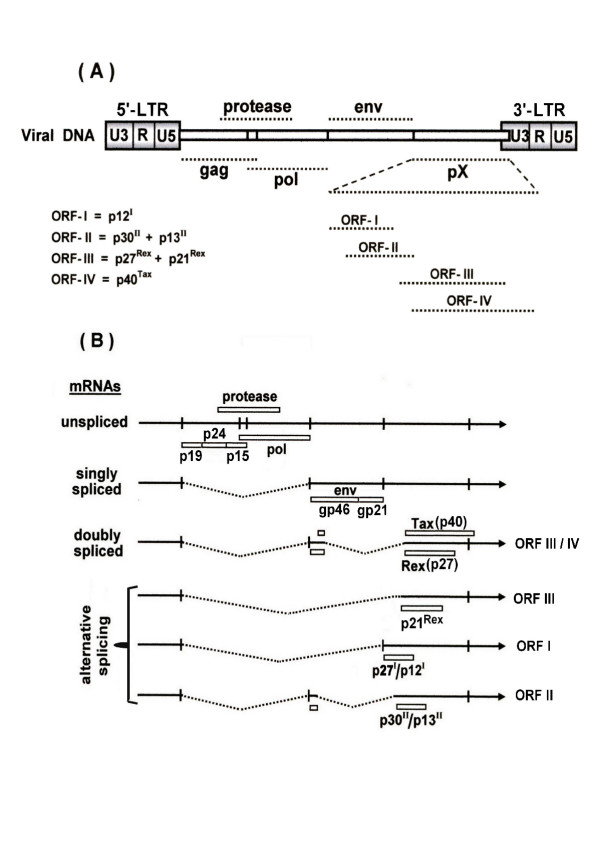
Schematic illustration of the HTLV-I genome organization (A) and its various mRNA species with their specific splicing and encoded protein products (B) (See the text for detailed explanation).

HTLV-1 is a complex retrovirus that, in addition to the two long terminal repeats (LTRs) and the gag, protease, pol and env genes, which are typical to most other retroviruses, its genome contains an additional region called pX, which resides between the env gene and the 3'-LTR,. This region includes four partially overlapping reading frames (ORFs), of which the most investigated ones are ORFs III and IV that encode for the viral regulatory Rex and Tax proteins respectively (see illustration in Fig. 1A). The gag, protease and pol precursor polypeptide is translated from the full genomic length viral RNA, whereas the env precursor polypeptide is translated from a singly spliced viral RNA. These precursor polypeptides are cleaved into the mature functional proteins by the viral protease. Tax and Rex are translated from a doubly spliced viral RNA, using two alternative translational initiation codons as illustrated in Fig. 1B.

Tax is present predominantly in the nucleus due to its nuclear localization signal (NLS) residing at its amino terminus [20,21]. However a substantial portion of Tax is present also in the cytoplasm due to its newly identified nuclear export signal (NES) [22]. Tax, which acts as a dimer [23], was originally discovered as a transactivator of viral RNA transcription from a promoter located at the 5'-LTR [24], but later proved to modulate the synthesis or function of a wide range of cellular regulatory proteins [25-27]. Rex, on the other hand, acts to promote the export of the unspliced and singly spliced viral RNAs species from the nucleus to the cytoplasm [28] by binding to a Rex responsive element (RxRE) residing in the 3' R region of the viral RNA [29]. In addition, there are some indications that Rex may also inhibit splicing and degradation of the viral RNAs [30]. Thus at high level of Rex there is a preferential export of the gag-protease-pol- and of the env-encoding RNA species and low export of the Tax/Rex-encoding RNA. This leads to a decline in the level of Rex and Tax proteins and consequently to a reduced viral RNA transcription. As a result, the Tax/Rex-encoding RNA is preferentially exported from the nucleus. In this manner Rex maintains these different RNA species at an optimal balance required for the virus production. Consistent with this notion Ye et al. [31] have shown that cells harboring proviral DNA with defective Rex reading frame produce high level of the doubly spliced tax/rex encoding mRNA and high level of functional Tax protein, but low level of p19 Gag protein and undetectable Rex protein. An alternatively spliced RNA encodes for another protein from ORF III, termed p21^Rex^, but its biological function is unclear [32].

More recently interest has been focused also on ORF I that encodes for p12 and p27 and ORF II that encodes for p13 and p30 proteins [33]. In contrast to Tax and Rex, which are encoded by a bicistronic pX mRNA formed by double splicing of the viral RNA [21,34,35], the other four accessory proteins are encoded by different pX mRNAs formed by alternative splicing events [33,36]. Pique et al. [37] have detected CTL activity in HTLV-I infected individuals against specific peptides from each of these ORF I and ORF II proteins, indicating that each of them is produced natural human infections. The functional role of these accessory proteins is not completely clear yet. Certain studies have demonstrated that deletions within frame I and II do not affect the replication and infectivity of HTLV-1 [36] nor its capacity to immortalize primary T-cells [36,38]. In contrast, by using molecular HTLV-1 clone, the group of Albrecht and Lairmore has provided evidence for the critical role of these accessory proteins in the viral replication and pathogenesis [33]. It has been shown that ablation of frame I markedly reduced the virus ability to infect quiescent peripheral blood lymphocyte (PBLs) [39] and to replicate in a rabbit model [40]. The explanation suggested by these investigators for the discrepancy between theirs and the others' results regarding p12 is that the other groups examined the role of this protein in IL-2/mitogen-activated PBLs, whereas their own data indicate that p12 is required for HTLV-1 infection in quiescent PBLs, since when they added a mitogen and IL-2 to their cultures the p12-defective HTLV-1 clone became highly infective [33,41]. Notably, p12 localizes to the endoplasmic reticulum (ER) and is associated with two ER-resident proteins; calerticulin and calnexin. Calerticulin is a calcium-binding protein that participates in calcium signaling and linked to activation of the transcription factor nuclear factor of activated T-cells (NFAT) [42]. In this manner, p12 can activate the HTLV-1 DNA clone-harboring quiescent PBLs and provide the physiological requirements for its infectivity, or vice versa, mitogen/IL-2 activation of the PBLs can override the deficiency imposed by the p12-defective clone. Since HTLV-1 targets quiescent T-cells in natural infection, these findings suggest an important role of p12 protein for the virus in vivo infectivity.

The frame II encoded p30 protein has been shown to localize to the nucleus and to function as a transcription factor. Transient transfection experiments have demonstrated that this protein can modulate the expression of various promoters and to activate HTLV-I LTR expression independently of Tax [43]. It was also shown to interact with the transcriptional co-activators CREB-binding protein (CBP) and p300 [44]. Together, these and other data indicate that p30 may account for the activation of several genes in HTLV-1 infected cells [44] and play an important role in the virus replication [45] and maintaining high viral load in in-vivo infection [33,39,46]. In contrast, a recent study by Nicot et al. [47] have shown that p30 rather inhibits HTLV-I expression by binding to the tax/rex-encoding doubly spliced viral RNA and retaining it in the nucleus. In this manner p30 prevents the synthesis of Tax and Rex proteins and interferes, thereby, with the production of viral particles. Furthermore, high level of p30 has been found to interfere with Tax-induced activation of HTLV-I LTR [44]. In view of these data it has been suggested that by reducing HTLV-I expression high level of p30 protects the infected cells from the anti HTLV-I immune response and contribute, in this manner to the virus persistence [33]. The other frame II-encoded protein, p13 localizes in the mitochondria and alters its morphology and function [48]. This protein has been shown to be also essential for maintaining high viral load in rabbit [45,46]. It has been also demonstrated that p13 interferes with the phosphorylation of the guanine nucleotide exchanger Vav protein in T-cells [49].

Fig. 1 describes schematically the viral genome organization, its various mRNA species and the encoded proteins.

Since Tax protein is widely regarded as a key element in the HTLV-1 related leukemogenic process. We will discuss in the following sections the molecular activities and biological effects of Tax that seem to contribute to its oncogenic potential.

### Modulation of viral and cellular gene expression by Tax

#### Tax-mediated activation of CREB/ATF-dependent gene expression

**Figure 2:**
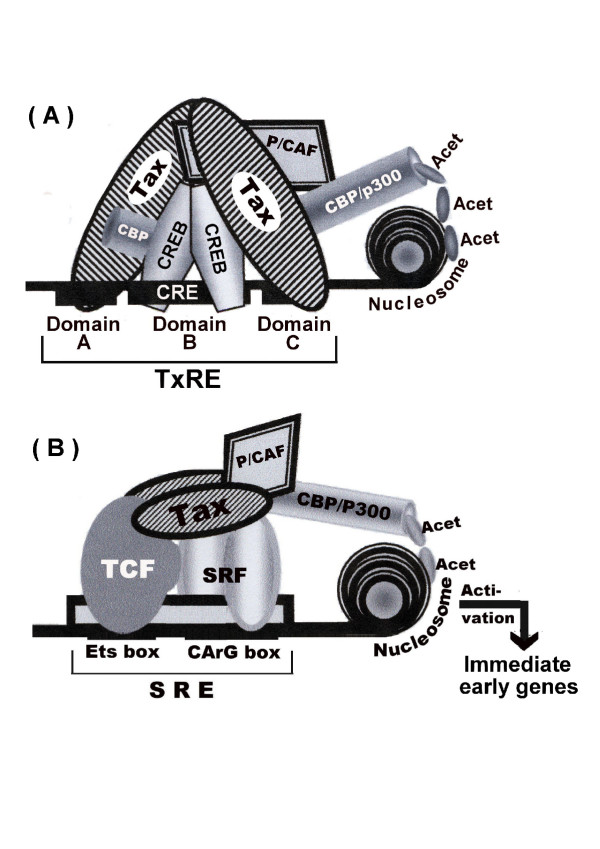
Schematic illustration of the DNA elements and the activator and co-activator proteins involved in Tax-induced transcriptional activation of (A) HTLV-I LTR and (B) SRF-dependent promoters (See the text for detailed explanation).

As noted before, Tax was initially discovered as a transactivator of the HTLV-1 gene expression [24]. It activates the viral LTR through three imperfectly conserved 21 bp repeats called Tax responsive elements (TxRE) [50], which contain a centered sequence TGACG(T/A)(C/G)(T/A) that is imperfectly homologous to the consensus cAMP responsive element (CRE; TGACGTCA) [51]. This element, which is also referred to as domain B of the TxRE, is flanked by a short G-rich stretch (AGGC) at its 5' side, termed domain A and a C-rich stretch (CCCC) at its 3' side, termed C domain C [27,51] (Fig. 2A). Although several basic leucine zipper (bZIP)-containing proteins, belonging to the CRE-binding/activating transcription factor (CREB/ATF) family, can bind to this viral CRE [52] only few of them can efficiently mediate the Tax-induced transactivation of HTLV-1 LTR [53-56]. A recent investigation of the effect of negative transdominant constructs against various bZIP proteins of this family has provided evidence that CREB is the most prominent factor that cooperates with Tax in activating HTLV-1 LTR expression [53]. Numerous earlier studies have demonstrated that in the absence of Tax, CREB forms unstable complex with the viral CRE, whereas Tax acts to stabilize this complex. By interacting with the bZIP region of CREB Tax enhances CREB dimerization and increases, thereby, its affinity to CRE [54,57-59]. This Tax-CREB-TxRE complex is further stabilized by direct binding of Tax to domains A and C of the TxRE through its N-terminus [60,61] (Fig. 2A). This stabilized binding enables Tax to recruit to the ternary Tax-CREB-TxRE complex the co-activators CREB binding protein (CBP) and its homologous protein p300 by binding to their KIX domain through its kinase-inducible domain (KID) [62] and the p300/CBP-associated factor (P/CAF), which binds through it carboxy terminus to a distinct site located around amino acid 318 to 320 of the Tax protein [63]. These three co-activators exert their effect by histone acethylation, which induces chromatin conformational modification at the site of the target promoter and facilitates, thereby, the interaction of the enhancer-bound transcriptional activators with the TATAA box-associated basal transcriptional factors [27] (Fig. 2A). Interestingly, however, Jiang et al. have shown that P/CAF can bind Tax without CBP or p300 and enhances its stimulatory effect on HTLV-1 LTR transcriptional expression independently of histone acetylation [63]. In contrast, several other studies have indicated that CREB2 (called also ATF-4), a member of another bZIP protein family, plays a more central role in Tax activation of HTLV-1 gene expression. These studies show that while in the absence of Tax, CREB can activate HTLV-1 LTR expression only if phosphorylated by protein kinase A (PKA), CREB2 can markedly activate the viral LTR without phosphorylation and that this protein mediates a much stronger activation of the viral LTR by Tax than CREB does [64-66].

Of particular note are also the recent observations that when two copies of the TxRE are placed upstream to TATAA boxes from HTLV-1 LTR or from other promoters, the strongest activation by Tax is detected with the TATAA box of the HTLV-1 LTR, indicating that this TATAA box contains a specific Tax responsive element. Furthermore, these studies have also revealed that beside of the enhancing effect Tax on the association of the TATAA-box binding protein (TBP) to the TATAA site, Tax has an additional stimulatory effect that is directed towards a step occurring after the assembly of the basal transcriptional factors onto the TATAA box [53].

Many cellular genes contain in their promoters a consensus CRE element and are activated by signals that elevate the cellular cAMP level. The elevated cAMP activates PKA to phosphorylate CREB which, in turn, binds to CRE and to CBP/p300. However, there is a substantial controversy on whether Tax can activate only the viral CRE in its context with the CG-rich flanking domains in the viral LTR [25,61], or also CRE located in cellular promoters [67,68]. In addition, there are data demonstrating that Tax uses the CREB/ATF factors to repress the expression of certain genes, like the cyclin A [69], p53 [70] and c-myc [71]. This CRE-dependent effect of Tax on such cellular genes may contribute to the initiation of an oncogenic process by impairing the cell cycle and growth control.

#### Tax mediated activation of SRF-dependent gene expression

HTLV-1 infected and Tax-expressing T-cell lines display increased expression of immediate early genes such as c-Fos, c-Jun, JunB, JunD and Fra-1, which are components of the dimeric transcription factors AP1, Egr-1 and Egr-2 [72], fra-1 [73], Krox-20 and Krox-24 [74]. Formation of these transcription factors is normally activated by the serum responsive factor (SRF) in response to various mitogenic signaling agents like serum, lysophosphatidic acid (LPA), lipopolysaccharide (LPS), 12-O-tetradecanoylphorbol-13-acetate (TPA), cytokines and tumor necrosis factor-α (TNFα). SRF acts through an SRF responsive element (SRE) residing in the promoters of these genes [75]. The SRE region actually contains two binding sites; a CArG box [CC(A/T)_6_GG], and an upstream Ets box [GGA(A/T)]. After binding to the CArG box, SRF protein interacts with the ternary complex factors (TCFs), which consequently bind to the upstream Ets box. In addition, SRF requires for its transcriptional activity the CBP/p300 and p/CAF co-activators [76].

Tax activates these immediate early genes by interacting with SRF [77,78] and with TFCs, CBP/p300 and P/CAF [76] (Fig. 2B). Moreover, AP-1, which is highly expressed in HTLV-1 infected T-cells [79], regulates the expression of multiple genes essential for cell proliferation, differentiation and prevention of apoptosis [80], so that by activating SRF, Tax can also indirectly induce a wide variety of such cellular genes. Thus, constitutive activation of such genes in HTLV-1 infected T-cells independently of specific external signals might be a trigger for initial steps in the oncogenic transformation of HTLV-1 infected T-cells in culture as well as in human infection.

#### Tax-mediated activation of NF-κB-dependent gene expression

A substantial part of Tax oncogenic potential is attributed to its ability to activate transcription factors of the NF-κB family, since these factors regulate the expression of numerous cellular genes [81] associated with diverse biological processes, such as embryonic development, immune and inflammatory responses, cell growth, apoptosis, stress responses and oncogenesis [25,82-84]. The NF-κB factors are functionally related to the c-Rel proto-oncogene and include the p50(NF-κB1), p52(NF-κB2), p65(RelA), RelB and c-Rel proteins, which act in various combinations of homo- and heterodimers displaying distinct specificities. They share a common domain of 300 amino acids, termed Rel homology domain (RHD), which is involved in their dimerization, DNA binding and nuclear localization. The p65:p65 and p65:p50 κB are the most prominent dimers involved in NF-κB-dependent transcriptional activation, whereas the p50:p50 dimer is rather inhibitory [85].

**Figure 3:**
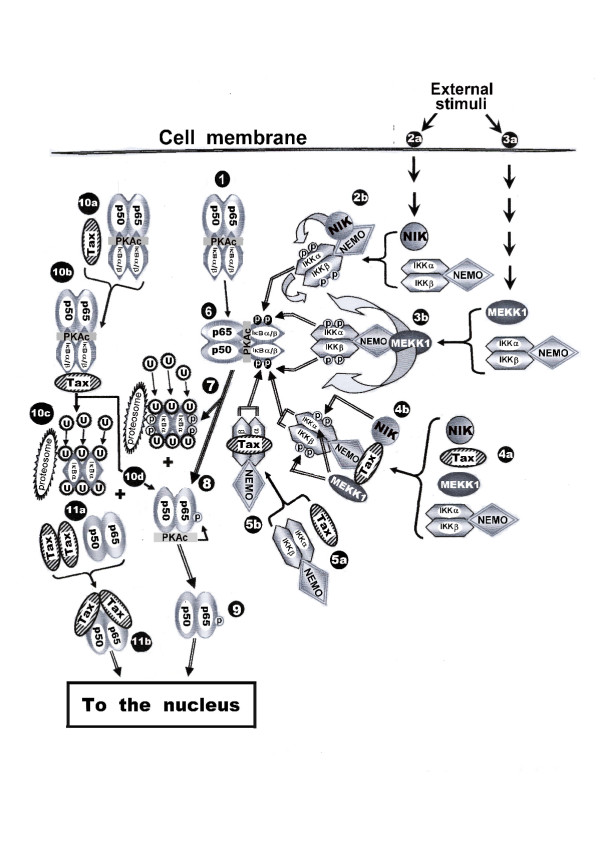
Schematic illustration of the factors and the molecular interactions associated with the release the NF-κB factors from their IκB inhibitors in the cytoplasm by external signaling stimuli and by HTLV-I Tax (See the text for detailed explanation).

In non-activated state NF-κB factors are trapped in the cytoplasm, tightly associated with inhibitory proteins called IκBs, primarily with IκBα and IκBβ. These inhibitors contain ankyrin repeats through which they bind to the RHD of the NF-κB factors and mask their nuclear localization signal (NLS) [86]. In addition, these complexes contain the catalytic subunit of protein kinase A (PKAc) which binds in the cytoplasm to both IκBα and IκBβ and is held there in an inactive state [87] (see illustration in Fig. 3 No. 1). NF-κB factors are activated in response to a wide variety of inflammatory cytokines and mitogens, such as TNF-α, IL-1, IL-6, IL-8, GM-CSF, bacterial lipopolysaccharide (LPS) and stress-inducing factors [81,83,84] (see Fig. 3, No. 2a and 3a). This activation proceeds in two phases, one taking place in the cytoplasm and the other in the nucleus.

The cytoplasmic phase includes phosphorylation of IκBα on serine32 and serine36 and of IκB on serine19 and serine23 (Fig. 3, No. 6), which is followed by their ubiquitination and subsequent proteosomal degradation [88] (Fig. 3, No. 7). The release from IκBs, activates the associated PKAc, which phosphorylates the free p65(RelA) factor at its serine276 (Fig. 3, No. 8). As will be discussed later in more details, this phosphorylation is essential for the transcriptional activity the p65(RelA)-containing dimers [87]. In addition, degradation of the IκBs releases the sequestered NF-κB dimers to translocate to the nucleus [88] (Fig. 3, No. 9). The phosphorylation of IκBs is carried out by an IκB kinase (IKK) complex comprised of two catalytic subunits, IKK and IKK and a regulator subunit, IKK which is called also NF-κB essential modulator (NEMO) [89,90] (Fig. 3, No. 2a and 3a). IKKα and IKK share a 52% amino acid identity and a similar domain structure that includes amino-terminal kinase domain, a dimerization leucine zipper domain, and helix-loop-helix motifs, which are involved in regulating their kinase activity [89,90].

**Figure 4:**
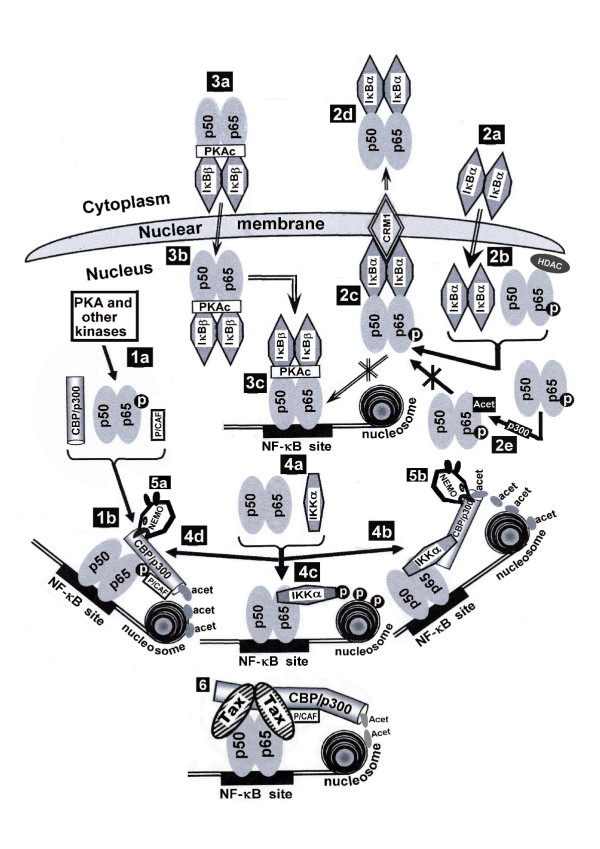
Schematic illustration of the factors and molecular interactions occurring in the nucleus which are involved in regulating the transcriptional competence of the NF-κB factors after reaching the nucleus and the function of HTLV-I Tax in this regulation (See the text for detailed explanation).

The phosphorylating function of the IKK complex is activated by upstream kinases such as the NF-κB inducing kinase (NIK) (Fig. 3, No. 2b), the mitogens-activated protein kinase/ERK kinase kinase-1 (MEKK1) (Fig. 3, No. 3b) and certain other signal-activated kinases [91]. NIK phosphorylates mainly the IKKα subunit (Fig. 3, No.2b), whereas MEKK1 activates both IKKα and IKKβ [92] (Fig. 3, No. 3b). Activation of IKKα results from its phosphorylation at serine176 and serine180, whereas IKK is activated by its phosphorylation at serine177 and serine181 [93,94]. Despite their high homology, IKKβ is much more active than IKKα in phosphorylating the IκBs [93,95,96]. This predominant activity of IKKβ over IKKα may be partially explained by the observation that in addition to the phosphorylation of IKKβ by MEKK1, IKKβ is directly phosphorylated also by IKKα, [97,98] (Fig. 3, No. 2b). A recent study has suggested an additional function for IKKα by showing that p65(RelA) needs to be phosphorylated by this kinase at serine536 in order to be transcriptionally active [99]. The third subunit, IKKγ/NEMO is devoid of kinase activity. Its role is to serve as a universal scaffold which connects between the two catalytic IKK subunits and their upstream activating factors into a large IKK complex [100,101] (Fig. 4, No. 2b and 3b). Iha et al., [102] have shown that these various factors assemble to the IKK complex through different domains of the IKKγ/NEMO protein, which could be selectively inactivated, thus attenuating certain NF-κB activating signals without affecting others.

**Figure 5:**
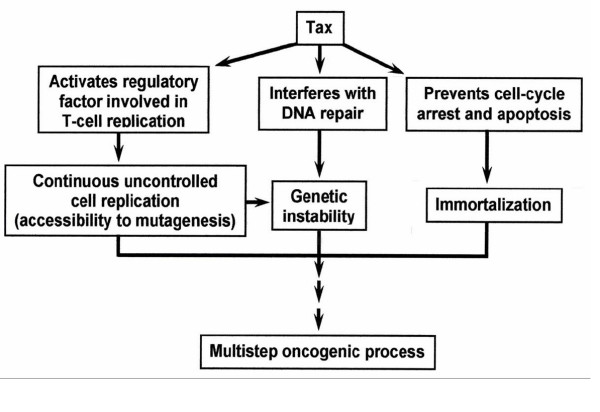
Schematic presentation of Tax biological effects which contribute to its oncogenic potential.

Recently, much interest has been attracted to the nuclear regulation of the NF-κB transcriptional competence. It has been shown that after reaching the nucleus p65(RelA) can bind the CBP/p300 and P/CAF coactivators which are essential for the transcriptional competence of p65(RelA):p65(RelA) and p65(RelA):p50 dimers [103]. This binding depends on p65(RelA) phosphorylation at serine276 by PKA and certain other signal activated serine kinases [85,87,104-108] (see illustration in Fig. 5, No. 1a and 1b). This phosphorylation is blocked by an NF-κB-inducible protein termed SINK, which binds to p65(RelA). This binding does not affect the nuclear localization of p65(RelA), nor its binding to the target DNA sites. Instead, by inhibiting p65(RelA) phosphorylation SINK prevents its association with the CBP/p300 and P/CAF co-activators, thus creating a negative feedback control of p65(RelA) transcriptional activity [109]. Another inhibitor protein, called RelA-associated inhibitor (RAI), has been identified in the nucleus of certain cell types where it can interact with p65(RelA) and inhibit its transcriptional activity by blocking its DNA binding. It has been proposed that this protein provides an alternative cell-type specific control of NF-κB-dependent gene expression [110].

In addition to its cytoplasmic inhibitory function IκBα plays an important regulatory role in the nucleus too. IκBα has an NLS signal which enables its translocation to the nucleus where it is protected from the signal-induced degradation described above [111]. Within the nucleus IκBα binds to the nuclear p65(RelA) and abrogates its transcriptional activity by inhibiting its DNA-binding [112]. IκBα has also a nuclear export signal (NES) which mediates the export of the p65(RelA):IκBα complex back to the cytoplasm via its interaction with the nuclear exporting protein CRM1 [113] (see Fig. 4, No. 2a, 2b, 2c and 2d). It has been proposed that as long as the signal-induced cytoplasmic degradation of the NF-κB-associated IκBα is active, induction of corresponding NF-κB-dependent gene expression can keep going on, whereas upon termination of this signal the export of the p65(RelA):IκBα complex from the nucleus may serve as an immediate terminator of this gene expression. However, the nuclear association of IκBα with p65(RelA) has been noted to depend on p65(RelA) acetylation status. The nuclear p65(RelA) can be acetylated by p300 and this acetylation avoids the binding of p65(RelA) to IκBα, thus preserving its transcriptional activity [114]. On the other hand, the nuclear p65(RelA) can bind to specific isoforms of histone deacetylase (HDAC) which deacetylate it and inhibit, thereby, its transcriptional activity by facilitating its association to IκBα [115]. (see Fig. 4. No. 2e). In contrast to this nuclear IκBα function, it has been noted that signals imposing persistent NF-κB activation, do so by enhancing the level of unphosphorylated IκBβ, which binds to p65(RelA) in the cytoplasm without masking its NLS or interfering with its DNA binding [116] (Fig. 4, No 3a, 3b and 3c). It has been proposed that under such conditions IκBβ escorts p65(RelA) to the nucleus, where it protects it from the inhibitory effect of the nuclear IκB and maintains, in this manner, a persistent NF-κB transcriptional activation [116].

IKK has also been found to have an important role in the nucleus (Fig. 4, No 4a) where it seems to affect the NF-κB transcriptional activity in several different ways. In one study the nuclear IKKα has been shown to bind CBP and p65(RelA) and to recruit, in this manner, the CBP co-activator to NF-κB-responsive promoters, where it acetylates histone H3 and facilitates, thereby, the expression of these promoters [117] (Fig. 4, No. 4b). Another study has shown that the nuclear p65(RelA)-associated IKKα stimulates the NF-κB-responsive promoters by directly phosphorylating histone H3 with its kinase activity [118] (Fig. 4, No 4c), and a third study has demonstrated that the nuclear IKKα phosphorylates the nuclear p65(RelA) and facilitates, thereby, its association with CBP/p300 [99] (Fig. 4, No 4d).

IKKγ/NEMO too has been noted to translocate to the nucleus where it regulates the NF-κB transcriptional activity by competing with the nuclear p65(RelA) and IKKα for CBP/p300 [119] (Fig. 4, No. 5a and 5b correspondingly).

In contrast to the transient NF-κB activation by external signals, NF-κB factors are constitutively activated by HTLV-1 Tax protein in Tax-expressing and HTLV-1-infected cells. Reported studies suggest that Tax may exert this activation in three ways: **a) **The most widely accepted concept is that Tax associates with the IKK complex through the adaptor IKKγ/NEMO subunit. Tax also binds to the upstream kinases, MEKK1 and NIK and enhances their kinase activity. In this manner Tax connects these activated kinases to IKKγ/NEMO and recruits their kinase activity to phosphorylate IKK and IKKβ [25,102,120-122] which, in turn, phoshphorylate IκBα and IκBβ (see Fig. 3, No. 4a, 4b and 6). A recent study have proposed that IKKγ/NEMO assembles into the large IKK complex as a homodimer or homotrimer and that its binding to Tax enhances its oligomerization [123]. **b) **Tax can bind directly to IKK and IKK and activates their kinase activity independently of their phosphorylation by the upstream signal-induced kinases [124] (Fig. 3, No. 5 and 6), **c) **Tax can bind directly to the IκBs and induce their proteosomal degradation independently of their phosphorylation by IKK [90,125] (Fig. 3, No. 10a, 10b and 10c). Thus, Tax induces phosphorylation-dependent or independent degradation of both of the IκBs and enables, thereby, the nuclear translocation of the released NF-κB factors independently of exogenous signals (Fig. 3, No. 9).

A number of studies indicate that the nuclear Tax plays an important role in establishing the transcriptional activity of the NF-κB factors reaching to the nucleus. Bex et al. [126] have demonstrated that the nuclear Tax localizes in transcriptionally active structures containing the NF-κB factors p50 and p65(RelA), RNA polymerase II, nascent RNA and splicing factors. Other studies have shown that Tax physically binds to the NF-κB factors p65(RelA) [127], c-Rel [127], p50 [128] and p52 [129] and enhances, thereby, their dimerization [130], which is essential for their binding to the NF-κB responsive element in the target promoters [127,128]. Tax has noted also to associate with these factors when they are already bound to their DNA targets and facilitates their transcriptional activity [127,128]. In contrast, our own experiments, to be published elsewhere (manuscript in preparation), indicate that the binding of Tax to the free p65(RelA) factor occurs already in the cytoplasm and then the two proteins translocate to the nucleus together (see Fig. 3, No. 11a and 11b). In addition, it has been demonstrated that by its ability to bind the NF-κB factors [127-129] on one hand and the CBP/p300 [62,131] and P/CAF [63] co-activators on the other hand, Tax recruits these co-activators to the NF-κB factors independently of the above mentioned serine276 phosphorylation on p65(RelA) (see illustration in Fig. 4, No 6). However, a recent study has indicated that in order to be transcriptionally active, p65(RelA) needs to be phosphorylated by IKKα at serine536 even when activated by Tax [99]. This phosphorylation is mediated by Tax [99] through its capacity to physically bind IKK and IKKβ and induce their kinase activity [124].

### Tax biological effects contributing to its oncogenic potential

#### Enhancing T-cell proliferation

There is ample of literature, reviewed in ref. [2,3,21,132,133], which demonstrate a modulation of expression of a wide range of cellular genes by Tax. cDNA profile analyses have detected several hundreds of Tax modulated cellular genes [82,134]. Some of them are directly involved in activation of T-cells proliferation, such as interleukin 2 (IL-2) [135] and the α subunit of its receptor (IL-2Rα) [136], which together establish an autocrine loop [137], IL-15 [138] and its receptor (IL-15R) [139], granulocyte-macrophage colony stimulating factor (GM-CSF) [140], tumor necrosis factor-α (TNF-α) [141], the MAD1 [142] and others [21]. Tax also activates cyclin D2 [143], cyclin D3 [144] and cdk6 [145], which are involved in the cell cycle progression, and inactivates p16^INKA4 ^[146] which acts to restrain excessive cycle progression. In addition, Tax affects the functions of many regulatory proteins by physical binding to them. A recent protein profile analysis has revealed that Tax can form complexes with 32 different proteins. Many of them belong to the signal transduction and cytoskeleton pathways and transcription/chromatin remodeling [147]. Constitutive deregulation of such regulatory factors in HTLV-1 infected T-cells can set the cells into uncontrolled continuous proliferation. Induction of such a continuous proliferation of mature T-cells is likely one of the first steps in the initiation of the ATL leukemogenic process since it renders the cells more accessible to spontaneous and exogenously induced mutagenesis.

### Induction of genetic instability

#### Enhancing mutagenesis via telomerase inhibition

Telomeres are specialized nucleoprotein structures located at the ends of each chromosome. In human they consist of up to 15 kb long double stranded tracts of tandem TTAGG repeats, ending with a 3' single-stranded overhangs and are associated with a number of functional proteins [148]. These structures prevent chromosomes from fusing end-to-end with each other on one hand and protect them from degradation by exonucleases on the other hand. They also enable the cells to distinguish between ends of broken DNA and natural chromosomal ends and prevent these natural ends from initiating DNA damage-specific checkpoint or repair cascades [148]. Telomeres are formed by telomerase, which are present in germ and embryonal cells and in many cancers but not in normal adult somatic cells [149]. Hence, in the absence of telomerase activity the telomeres of normal somatic cells are progressively shortened in each cell division until they reach a critical length, at which point the cells enter a quiescent viable state and are subsequently eliminated by apoptosis. However, the same shortening process may abrogate the telomere's protective effects and allow, thereby, end-to-end chromosomal fusion that forms dicentric and multimeric chromosomal structures. Such structures can break during mitosis at variable points, resulting in aneuploidy and extensive non-reciprocal chromosomal translocations and rearrangements. This chromosomal instability can lead to accumulation of various mutations, including such that inactivate important checkpoints or induce a telomere-restoring mechanism, which may result in immortalization and carcinogenesis of the cells [150]. This implies that telomerase inactivation may, actually, play an important role in tumor initiation. Consistent with this notion many established human cancers maintain stabilized telomere length either due to mutational telomerase reactivation [149], or activation of other alternative mechanisms [151].

Of note in this context is, that unlike other types of human leukemia and lymphoma, ATL cells display numerous unique chromosomal aberrations [152,153] resembling those resulting from telomere dysfunction, frequently seen in solid tumors [149]. Moreover, HTLV-1 Tax has been recently found as capable of inactivating telomerase in a variety of cells [154], suggesting that telomerase inhibition in infected cells of HTLV-1 carriers might be one of the mechanisms by which Tax initiates the ATL-related leukemic process. This possibility is supported by data demonstrating that infection of primary peripheral blood T-lymphocytes with HTLV-1 results in an initial decline of the cell viability which parallels with reduction in telomerase activity and that this decline is subsequently followed by an outgrowth of selected immortal survivors displaying increased telomerase activity [155]. Additional support comes from the close correlation observed between telomerase activity in ATL cell and the clinical stage of the disease. Leukemic cells of acute ATL patients display the highest telomerase activity, whereas patients with less severe clinical stage, whose leukemic cells elicit high telomerase activity, were noted to rapidly progress to the acute form, suggesting that the increased telomerase activity is not a side result of the acute ATL conditions, but rather one of the causes leading to this stage [156].

#### Interference with DNA repair

As noted above, HTLV-1 infected T-cells show high frequency of chromosomal abnormalities [152,153]. The first indication that Tax is associated with cellular genetic aberration came from the observation that Tax represses the expression of polymerase-β which is involved in DNA repair [157]. This notion was later substantiated by demonstrating the capacity of Tax to enhance mutation rate [158] and other types of genetic instability [159] via impairing the chromosomal segregation fidelity and interfering with several modes of DNA repair such as the, mismatch repair (MMR), base excision repair (BER) and nucleotide excision repair (NER) [160-165]. Particular interest has been focused in the last few years on Tax interference with NER, since this mode of repair is regarded as a major mechanism of maintaining the genome stability and its abrogation has been linked to increased cancer incidence [166]. However, there are several unresolved questions regarding the role of some factors in these DNA repair pathways. For example, Tax has been shown to enhance the expression of PCNA [167], an essential cofactor of DNA polymerase-δ and , which are involved with DNA replication and repair [168]. Mutagenesis analysis have suggested that the ability of Tax to stimulate PCNA correlates with its ability to inhibit NER [162]. Based on this observation it has been proposed that the increase of PCNA molar ratio over polymerase-δ interferes with the DNA repair activity of this polymerase without affecting its function in DNA replication [160,162]. However, this explanation seems to over-simplify the complex role of PCNA in coordinating polymerase-δ activities between DNA replication and DNA repair. Of note in this context is that moderate levels of p53 also stimulates PCNA [169], but yet NER is rather enhanced in these conditions [161]. Another explanation has been based on p53 ability to elevate the level of p21^WAF-1 ^which binds to PCNA [170]. It has been proposed that this binding of p21^WAF-1 ^directs PCNA towards inhibition of DNA replication without affecting NER [168] and that Tax can prevent this pathway by its capacity to inhibit p53 transcriptional activities [70,171,172]. However, we [173] and others [174] have shown that Tax also elevates p21^WAF-1 ^and therefore, should accordingly, be anticipated to enhance NER rather than to inhibit it. Furthermore, other studies have demonstrated that p21^WAF-1 ^is not needed for NER [175] or may even inhibit it [176]. Therefore, more intensive studies are needed to resolve these conflicts. Of note however, p53 has been found to act also at the early damage-recognition step of NER [177]. It would be interesting to find out whether Tax directs its inhibitory effect towards this early step of NER.

In addition, p53 has been proved to be also directly involved in BER [178], suggesting that Tax interference with BER [164] might also be exerted through its inhibitory effect on p53 function. At any rate, this genetic destabilization by Tax is certainly an important element of Tax oncogenic potential.

#### Inhibition of topoisomerase I

Topoisomerase I (Topo-I) is involved in DNA synthesis and maintenance of the genome stability by participating in DNA repair and chromosome condensation. It alters DNA topology by transiently breaking one strand of the DNA, passing the other strand through the break and finally resealing the break [179]. Tax has been found to bind to topo-I and inhibit its activity [180], whereas Topo-I activity has been shown to be stimulated by p53 [181]. Thus Tax may interfere with Topo-I activity also through its effect on p53. This might be another way for Tax to destabilize the cellular genome, but more intensive investigation is required to further substantiate this possibility.

#### Tax-mediated protection of HTLV-1 infected T-cells from stress-induced cell cycle arrest and apoptosis

All the above effects of Tax which enhance mutations and other chromosomal aberrations and interfere with DNA repair should be expected to induce cell cycle arrest or apoptosis. This, in turn, should prevent further progression of the leukemogenic process in the infected cells, unless such cells can, somehow, escape the cell cycle arrest and apoptosis. There is a substantial controversy over the influence of Tax on the cell response to stress insults. While many studies have demonstrated that Tax protects cells from stress-induced cell cycle arrest or apoptosis [182-186], others have shown that it enhances the cell sensitivity to these stress-induced effects [186-190]. Indeed, cDNA microarray analysis of HTLV-1 Tax expressing cells exposed to DNA damage stress signal revealed elevated expression of pro- as well as of anti-apoptosis genes [191]. However, results from our [182] and other laboratories reviewed in ref. [186,191], suggest that in HTLV-1 producing T-cells the anti-apoptotic effects of Tax override its potential pro-apoptotic effects. Besides, Tax has been shown to suppress a wide range of factors participating in the apoptosis cascade on one hand and to stimulate factors acting as apoptosis inhibitors on the other hand [144,185,192,193]. A possible explanation for the above noted controversy is that in most of the studies presenting Tax pro-apoptotic effect, Tax was over-expressed through highly potent promoters. Excessive levels of Tax may, reasonably, sensitize the cells to apoptosis. However, these experimental conditions do not reflect the situation in HTLV-1 expressing T-cells, in which Tax cannot exceed the optimal level required for its replication due to the Rex-mediated fine regulation of the balance between the viral RNA species encoding for the gag, pol, prot and env proteins and those which encode for the Tax and Rex proteins [28,29] described earlier in this review. In addition, Tax has been shown to enhance the cell cycle progression and to release cells from stress-induced cell cycle arrest [26,145,194].

#### Experimental models for Tax oncogenicity

Numerous studies have been focused on investigating Tax oncogenic potential in cultured cells and animal models. Most of them used plasmids expressing w.t. Tax or various Tax mutants under the control of HTLV-1 LTR or other different promoters. It was only few years ago that an infectious clone of the entire HTLV-1 genome was constructed and appropriate cell culture techniques were developed to introduce this clone into human primary PBLs. This clone was found as capable of propagating in cultured mammalian cells and to transform primary human T-lymphocytes [195-197]. This clone opened an opportunity to study the oncogenic potential of Tax and of various Tax mutants in the context of the entire viral genome. Some of the studies to be discussed in this section have been performed with such clones.

#### Transformation of rodent cells

Tax has been shown to induce neoplastic transformation of the rat fibroblast Rat-1 [198-203] and the mouse fibroblast NIH/3T3 [201] cell lines. Tax has been also shown to cooperate with the ras oncogene in transforming primary embryo fibroblasts [203]. Transformation was determined by formation of foci of morphologically transformed cells, colony formation in soft agar and tumor formation in nude mice. Several mechanisms have been proposed to mediate the transformation of Rat-1 cells: 1) Involvement of NF-κB [199], 2) involvement of CREB/ATF [68], 3) involvement of phosphoinositide-3 kinase-PKB/Akt [202] and 4) Stimulation of p21^WAF-1 ^which prevents apoptosis and enhances the replication of the transformed cells [204]. The cooperation of Tax with ras in transforming primary embryo fibroblasts is postulated to be mediated by SRF through the CArG elements of SRF responding genes [198]. It should be emphasized, however, that maintenance of this transformation phenotype requires the continuous presence of active Tax and that no genetic mutation can be identified in these transformed cells [200]. Therefore, the process leading to this transformation is unlikely to reflect the entire pathway leading to ATL because the leukemic ATL barely express Tax [3] and they are characterized by intensive chromosomal aberration [152,153,165]. For the most, this transformation may reflect only the very early steps of the initiation of the ATL process.

#### Immortalization and transformation of primary human T-lymphocytes

A closer insight into the ATL leukemogenesis has been gained through studies using primary human T-lymphocytes. Such experiments have revealed that after infection in culture with HTLV-1 or permanent transfection with Tax, primary human T-cells undergo two stages of cellular changes. In the first stage the cell become immortalized but still remain dependent on IL-2 for their growth [205]. This immortalization has been shown to result from Tax-induced stimulation of the G1 phase-specific cyclin-dependent kinases CDK4 and CDK6, increased expression of signal transduction genes like cyclin G1, c-fgr, hPGT [206] and p21^WAF-1 ^[204] and to be associated with mutations conferring increased telomerase activity [155]. Studies with different Tax mutants, deficient of CREB/ATF- or NF-κB-activation, have yielded conflicting results as to which of these two major regulatory pathways is involved in this Tax-mediated immortalization. While certain studies have shown that this immortalization depends on Tax ability to activate NF-κB [207,208], others have demonstrated that Tax mutants deficient of NF-κB activation still retain their capacity to induce this immortalization [209]. On the other hand, experiments with an infectious molecular clone of the entire HTLV-1 genome have shown that disruption of Tax ability to activate CREB results in preferential immortalization of CD8+ lymphocytes, rather than preferential immortalization of CD4+ lymphocytes seen with the wild-type infectious clone [210]. In addition, it has been found that disruption of Tax capacity to interact with CBP/p300 does not affect its immortalizing potential [195,210].

In the second stage few IL-2-independent clones of transformed cell emerge. Such transformed cells display an IL-2-independent constitutive activation of the IL-2 receptor (IL-2R) signaling pathway that includes the Janus kinases JAK1 and JAK3, and the signal transducers and activators of transcription STAT3 and STAT5, which are constitutively active in such cells [206,211-213]. Other studies have shown in such transformed cells a constitutive high expression of the growth factor independence-1 (Gfi-1) which is also involved in coffering their IL-2-independent growth [214]. In addition, intensive studies have been recently focused on factors participating in a negative regulation of the IL-2R associated pathway in HTLV-1 transformed T-cells. One of these factors is the SH2-containing tyrosine phosphatase SHP1. A gradual loss of this phosphatase has been noted to correlate with the progression of HTLV-1 infected primary T-cells from the immortalization (IL-2 dependence) to the transformation (loss of IL-2 dependence) stage [213,215]. Changes in other negative regulators of Jak/STAT/IL-2R pathway have been noted to vary between different transformed clones and, therefore, their role in acquiring the IL-2-indepence is unclear yet [213]. Also notable is that in contrast to the rodent cells, the HTLV-1 transformed primary human T-cells display high mutation rate [158] and other genetic aberrations [159,165,216]. Of particular interest in this context is the observation that exposure of HTLV-1 infected primary T-cells to carcinogens enhances a stepwise progression from their IL-2-dependent immortalized state to the autonomous transformed state [217]. Also interesting is the association noted between chromosome changes in such cells and their growth potential [216].

Many of the above changes observed in the HTLV-1/Tax immortalized and transformed primary human T-cells are quite analogous to those found in ATL cells. However, while fresh leukemic cells from ATL patients, as well as cell lines derived from these leukemic cells, are successfully engrafted in SCID mice and their leukemic infiltration to various organs is similar to that seen in ATL patients [218], primary human T-cells immortalized or transformed in culture by HTLV-1 infection, Tax transfection or intruding a molecular clone of the entire HTLV-1 genome, do not show such tumorigenicity in these mice [196,219]. This observation can be explained by postulating that during their progression through multiple selection steps in the infected patient the ATL leukemic cells accumulate selected genetic changes conferring their tumorigenic phenotype, whereas under culture conditions there is no selective pressure for preferential accumulation of such particular mutations.

#### Tumor induction in transgenic mice

Tax transgenic mice have been widely used as models for investigating the oncogenic effects of Tax in-vivo, hoping to get closer insight to the ATL leukemogenic process in human [220]. A wide range of different tumors have been described in such animals and it appears that the promoter used to express Tax determines at least partially the type of the developing tumors. Transgenic mice expressing Tax through HTLV-1 LTR were found to develop neurofibrosarcomas [221], mesenchymal tumors [222] or skeletal bone abnormalities [223], but not leukemias or lymphomas. Mice expressing Tax through the promoter of CD3ε were found to develop mesenchymal tumors at wound sites and salivary and mammary adenoma [224]. Only mice expressing Tax through the granzyme B promoter showed Tax expression in mature T-lymphocytes and developed large granular lymphocytic leukemia [225]. These studies suggest that Tax alone is capable of inducing tumors in various tissues, including lymphoid cells. A possible explanation for the failure of HTLV-1 LTR-Tax to induce leukemia in such animals may be provided by the observation that expression of this construct can be detected in various non-lymphoid organs like the brain, saliva glands, spleen, thymus, skin, muscle, bones and mammary glands [223,226] but not in the bone marrow [223]. It has been proposed that activation of HTLV-1 LTR expression in lymphoid cells requires the cooperation of the accessory proteins encoded by ORF1 and/or ORF II of the pX region with Tax protein. Therefore, Tax alone cannot activate the expression of the HTLV-1 LTR-Tax construct in these cells, whereas this cooperation is not needed in other organs. [3,33,226]

Various modes of tumor induction by the transgenic Tax have been noted so far. Hall et al. [224] have shown that the mesenchymal and the mammary adenomal tumor induced by the CD3ε-Tax transgene displayed high levels of apoptosis which is associated with high levels of Myc, Jun and p53. In contrast Portis et al. [227] have demonstrated a Tax mediated functional inactivation of p53 in the early stage of the large granular lymphocytic tumor formation by the granzyme B-Tax transgene and p53-inactivating mutations in a later stage of the tumor progression. Other studies have demonstrated the importance of Tax-mediated activation of NF-κB in the induction of both the lymphoid [228] and non-lymphoid [229] tumors.

As noted before, continuous Tax expression is required for maintaining the neoplastic phenotype of Rat-1 cells transformed by Tax in culture [200]. In contrast, suppression of Tax expression in transformed fibroblasts derived from tumors of Tax transgenic mice did not affect their growth rate and ability to form tumors in animals [230], indicating that Tax was involved only in the initiation of the in-vivo tumorigenic process, after which the cells continued to progress through several genetic changes rendering their neoplastic phenotype independent of Tax.

#### Conclusive comments about the pathways leading to ATL and TSP/HAM

**Figure 6:**
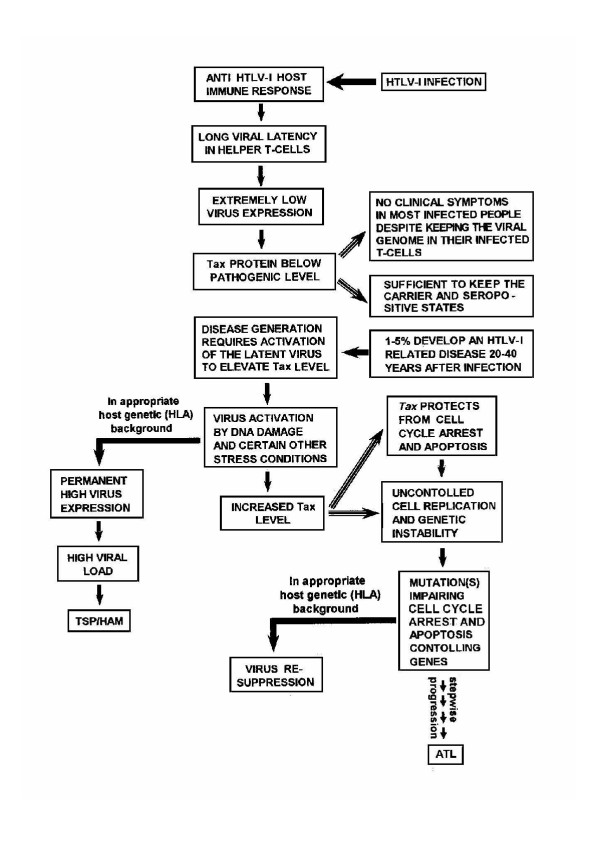
Schematic hypothetical flow of the events occurring between the initial infection with HTLV-I and ATL or TSP/HAM development (See the text for detailed explanation).

In view of the above described pleiotropic effects of Tax, which are summarized in Fig. 5, it is widely accepted that the viral Tax protein is a key element in ATL genesis [2]. This implies that generating this malignancy requires active viral gene expression in the infected T-cells of HTLV-1 carriers in order to keep Tax protein at an effective level. However, as noted before, shortly after establishing the host immune response against the viral antigens, HTLV-1 virus expression is kept very low and is the level of Tax. This low Tax level accounts, likely, the "carrier" state of the infected individuals by supporting a limited but continuous expansion of the infected CD4+ cells [17] and for their anti HTLV-1 seropositive states. However, this low Tax level, plausibly, is insufficient for exerting all the above described oncogenic effects leading to ATL. Therefore, over 95% of the infected individuals do not develop this malignancy, or other HTLV-1 related clinical disorders, during their entire life [2,3]. Thus, generating this malignancy would, plausibly, require activation of the dormant virus in order to elevate Tax to its oncogenic threshold. Our previous studies [182,231-233] indicate that this activation can be induced by a variety of stress agents which are widely present in the daily human surrounding. However, such agents normally induce also cell cycle arrest or apoptosis, which could be expected to prevent the subsequent progression towards ATL. This paradoxical conflict was resolved by our observation that Tax protects HTLV-1 producing human T-cells from stress-induced apoptosis [182], implying that the Tax protein, emerging after activation of the latent virus, can rescue the host cells from the stressed-induced apoptosis. After this activation step there may, actually, be a progression to either ATL or TSP/HAM. Intensive studies, reviewed in ref. [3,234], indicate that genetic factors of the host, mainly those associated with the HLA histocompatibily complex class I, are the major factors determining whether the progression will proceed towards ATL or TSP/HAM [235]. Of note is that TSP/HAM is characterized by high virus expression [236-238]. Such high virus expression is widely considered to be a predisposing factor for TSP/HAM development [239]. We [240] and others [235] discussed in details in earlier review articles, how this high virus expression accounts for most of the pathological and immunological manifestations of this syndrome and correlates with its severity [238]. On the other hand, no or very little Tax can be detected in the leukemic cells of ATL patients [3,12,13,16,234]. It has been proved that anti Tax CTLs mounted in these patients eliminate the rare cells with high Tax expression and keep, thereby, Tax at very low level [10,16]. This difference seems to be determined by the HLA type of the host [14]. It can be postulated that the immune response of people with HLA types of high risk for TSP/HAM, permits permanent high expression of the activated virus, whereas the immune response of people with other HLA types probably act to re-suppress the activated virus. Therefore, progression towards ATL can, presumably, proceed only if a mutation, that abrogates one of the important cellular checkpoints, occurs before the activated virus is re-suppressed. This speculation is supported by reports showing that the leukemic cells of most ATL patients carry one or more mutations which deregulate the formation or function of cellular factors associated with T-cell replication, cell cycle arrest or apoptosis, such as the IL-2 receptor [241], the JAK/STAT proteins [242], the growth factor independence 1 (Gfi) [214], p53 [243-245], p15^INK4B ^and p16^INK4A ^[246], p27^KIP1 ^[247], p16 (CDKN2) [248], pRb [249], surviving [193], Fas (Apo1/CD95) [250] and caspases [251]. Interestingly, the leukemic cells of most ATL patient are defective in the mitotic spindle checkpoint [252], which likely accounts for the frequent clastogenic and aneugenic chromosomal abnormalities detected in these cells [153]. However, no mutation in mitotic checkpoint genes has been identified in such cells [252]. Instead, the viral Tax protein has been shown, in one study, to inactivate the function of the mitotic spindle checkpoint protein MAD1 [142]. In another study the MAD1 and MAD2 checkpoint proteins, which normally reside in the nucleus, have been found in HTLV-1 infected cells to localize predominantly in the cytoplasm [252]. Since Tax is hardly detected in circulating ATL cells, it is rather unlikely to ascribe this checkpoint loss in the ATL cells to Tax activity. It seems more reasonable to speculate that this loss results from mutations in other genes which might indirectly affect the proper subcellular localization of these proteins. It is also reasonable to assume that this and the other mutations in the above mentioned regulatory genes are, most likely, acquired in the pre-leukemic stage during a certain time-gap when the cells are highly susceptible to mutagenesis. We like to propose that this time-gap is the time when Tax is still highly active in a large number of circular T-cells due to the putative activation of the latent virus. It is plausible to assume that the level of the anti Tax antibodies existing in latent HTLV-1 carriers is sufficient to repress Tax expression in the few high virus-expressing CD4+ cells [13] existing before activation of the latent virus and that existing level of anti Tax CTLs in such carriers is sufficient to eliminate these cells [10,16], but neither of these Immune response arms is sufficient to handle the overwhelming number of such high virus-expressing cells resulting from the virus activation. However, this situation is likely temporary and may plausibly last until the anti Tax antibodies and CTLs boosted to mount to a sufficiently higher level that can overcome this large number of high virus-expressing cells. This is the time-gap during which we thing that the pre-leukemic cells are most susceptible to mutagenesis and can acquire one or more of the above mentioned checkpoint-abrogating mutations. If, after the occurrence of such mutations, Tax expression is re-suppressed by the mounting level of the anti Tax antibodies and cells that still remain with high virus expression are eliminated by the mounting CTLs, this will not stop the remaining mutant cells to further accumulate additional mutations and progress towards ATL. This re-suppressed Tax expression will avoid exposure of the progressing cells to anti Tax CTLs. However, since the probability for such particular mutations to occur during this limited time-gap might be very low, it is quite possible that multiple episodes of such virus activation and re-suppression may occur in HTLV-1 carriers before progression to ATL can be turned on. Such flow of events, which is illustrated in Fig. 6, may explain why ATL usually develops after much longer clinical latency than TSP/HAM.

## Authors' contributions

Authour 1; (A.I) prepared the Fig.ures and together with author 2 (S-K.Y) covered the cited publications and prepared the draft of this review. Author 3 (A.M) designed the outlines of the review and together with the other two authors prepared the final version for submission. All authors read and approved the final manuscript.
